# Birth size and gestational age in opposite-sex twins as compared to same-sex twins: An individual-based pooled analysis of 21 cohorts

**DOI:** 10.1038/s41598-018-24634-2

**Published:** 2018-04-19

**Authors:** Aline Jelenkovic, Reijo Sund, Yoshie Yokoyama, Yoon-Mi Hur, Vilhelmina Ullemar, Catarina Almqvist, Patrik KE Magnusson, Gonneke Willemsen, Meike Bartels, Catharina EM van Beijsterveldt, Leonie H. Bogl, Kirsi H Pietiläinen, Eero Vuoksimaa, Fuling Ji, Feng Ning, Zengchang Pang, Tracy L Nelson, Keith E. Whitfield, Esther Rebato, Clare H. Llewellyn, Abigail Fisher, Gombojav Bayasgalan, Danshiitsoodol Narandalai, Morten Bjerregaard-Andersen, Henning Beck-Nielsen, Morten Sodemann, Adam D. Tarnoki, David L. Tarnoki, Syuichi Ooki, Maria A. Stazi, Corrado Fagnani, Sonia Brescianini, Lise Dubois, Michel Boivin, Mara Brendgen, Ginette Dionne, Frank Vitaro, Tessa L Cutler, John L. Hopper, Robert F. Krueger, Matt McGue, Shandell Pahlen, Jeffrey M. Craig, Richard Saffery, Claire MA Haworth, Robert Plomin, Ariel Knafo-Noam, David Mankuta, Lior Abramson, S Alexandra Burt, Kelly L. Klump, Robert F. Vlietinck, Catherine A. Derom, Ruth JF Loos, Dorret I. Boomsma, Thorkild I. A. Sørensen, Jaakko Kaprio, Karri Silventoinen

**Affiliations:** 1 Department of Social Research, University of Helsinki, Helsinki, Finland; 2 Department of Genetics, Physical Anthropology and Animal Physiology, University of the Basque Country UPV/EHU, Leioa, Spain; 3 Institute of Clinical Medicine, University of Eastern Finland, Kuopio, Finland; 4 Department of Public Health Nursing, Osaka City University, Osaka, Japan; 5 Department of Education, Mokpo National University, Jeonnam, South Korea; 6 Department of Medical Epidemiology and Biostatistics, Karolinska Institutet, Stockholm, Sweden; 7 Pediatric Allergy and Pulmonology Unit at Astrid Lindgren Children’s Hospital, Karolinska University Hospital, Stockholm, Sweden; 8 Department of Biological Psychology, VU University Amsterdam, Amsterdam, Netherlands; 9 Institute for Molecular Medicine FIMM, Helsinki, Finland; 10 Department of Public Health, University of Helsinki, Helsinki, Finland; 11 Obesity Research Unit, Research Programs Unit, University of Helsinki, Helsinki, Finland; 12 Endocrinology, Abdominal Center, Helsinki University Central Hospital and University of Helsinki, Helsinki, Finland; 13 Department of Noncommunicable Diseases Prevention, Qingdao Centers for Disease Control and Prevention, Qingdao, China; 14 Department of Health and Exercise Sciencies and Colorado School of Public Health, Colorado State University, Fort Collins, USA; 15 Psychology and Neuroscience, Duke University, Durham, NC USA; 16 Health Behaviour Research Centre, Department of Epidemiology and Public Health, Institute of Epidemiology and Health Care, University College London, London, UK; 17 Healthy Twin Association of Mongolia, Ulaanbaatar, Mongolia; 18 Graduate School of Biomedical and Health Sciences, Hiroshima University, Hiroshima, Japan; 19 Bandim Health Project, INDEPTH Network, Bissau, Guinea-Bissau; 20 Research Center for Vitamins and Vaccines, Statens Serum Institute, Copenhagen, Denmark; 21 Department of Endocrinology, Odense University Hospital, Odense, Denmark; 22 Department of Infectious Diseases, Odense University Hospital, Odense, Denmark; 23 Department of Radiology, Semmelweis University, Budapest, Hungary; 24 Hungarian Twin Registry, Budapest, Hungary; 25 Department of Health Science, Ishikawa Prefectural Nursing University, Kahoku, Ishikawa, Japan; 26 Istituto Superiore di Sanità - Centre for Behavioural Sciences and Mental Health, Rome, Italy; 27 School of Epidemiology, Public Health and Preventive Medicine, University of Ottawa, Ottawa, Ontario, Canada; 28 École de psychologie, Université Laval, Québec, Canada; 29 Institute of Genetic, Neurobiological, and Social Foundations of Child Development, Tomsk State University, Tomsk, Russian Federation; 30 Département de psychologie, Université du Québec à Montréal, Montréal, Québec, Canada; 31 École de psychoéducation, Université de Montréal, Montréal, Québec, Canada; 32 The Australian Twin Registry, Centre for Epidemiology and Biostatistics, The University of Melbourne, Melbourne, Victoria, Australia; 33 Department of Epidemiology, School of Public Health, Seoul National University, Seoul, Korea; 34 Department of Psychology, University of Minnesota, Minneapolis, MN USA; 35 Murdoch Childrens Research Institute, Royal Children’s Hospital, Parkville, Victoria, Australia; 36 Department of Paediatrics, University of Melbourne, Parkville, Victoria, Australia; 37 MRC Integrative Epidemiology Unit, University of Bristol, Bristol, UK; 38 King’s College London, MRC Social, Genetic & Developmental Psychiatry Centre, Institute of Psychiatry, Psychology & Neuroscience, London, UK; 39 The Hebrew University of Jerusalem, Jerusalem, Israel; 40 Hadassah Hospital Obstetrics and Gynecology Department, Hebrew University Medical School, Jerusalem, Israel; 41 Michigan State University, East Lansing, Michigan USA; 42 Centre of Human Genetics, University Hospitals Leuven, Leuven, Belgium; 43 Department of Obstetrics and Gynaecology, Ghent University Hospitals, Ghent, Belgium; 44 The Charles Bronfman Institute for Personalized Medicine, The Mindich Child Health and Development Institute, Icahn School of Medicine at Mount Sinai, New York, NY USA; 45 Novo Nordisk Foundation Centre for Basic Metabolic Research (Section of Metabolic Genetics), Faculty of Health and Medical Sciences, University of Copenhagen, Copenhagen, Denmark; 46 Department of Public Health (Section of Epidemiology), Faculty of Health and Medical Sciences, University of Copenhagen, Copenhagen, Denmark; 47 Osaka University Graduate School of Medicine, Osaka University, Osaka, Japan

## Abstract

It is well established that boys are born heavier and longer than girls, but it remains unclear whether birth size in twins is affected by the sex of their co-twin. We conducted an individual-based pooled analysis of 21 twin cohorts in 15 countries derived from the COllaborative project of Development of Anthropometrical measures in Twins (CODATwins), including 67,850 dizygotic twin individuals. Linear regression analyses showed that boys having a co-twin sister were, on average, 31 g (95% CI 18 to 45) heavier and 0.16 cm (95% CI 0.045 to 0.274) longer than those with a co-twin brother. In girls, birth size was not associated (5 g birth weight; 95% CI −8 to −18 and −0.089 cm birth length; 95% CI −0.202 to 0.025) with the sex of the co-twin. Gestational age was slightly shorter in boy-boy pairs than in boy-girl and girl-girl pairs. When birth size was standardized by gestational age, the magnitude of the associations was attenuated in boys, particularly for birth weight. In conclusion, boys with a co-twin sister are heavier and longer at birth than those with a co-twin brother. However, these differences are modest and partly explained by a longer gestation in the presence of a co-twin sister.

## Introduction

Birth weight is an indicator of foetal growth and predicts short-term survival of the newborn^1^. It is also an indicator of processes that influence long-term health; for example, birth weight has been inversely associated with adult mortality, especially cardiovascular mortality, and positively associated with the risk of cancer deaths^2^. It is well established that boys are born heavier than girls, in both singletons^3,4^ and twins^5–7^. However, there is an ongoing debate as to whether birth weight in twins is affected not only by their own sex but also by the sex of their co-twin. Dizygotic twinning rate has shown a steep increase since the 1980s in most industrialized countries, mainly due to the widespread use of *in vitro* fertilization and other fertility treatments^8,9^. Therefore, the study of the association between birth weight and the sex of the co-twin is of clinical and epidemiological interest.

Some studies have shown that boys from opposite-sex (OS) pairs are significantly heavier at birth than boys from same-sex (SS) pairs^10–12^, but this difference did not reach significance in other studies^13–16^. The greater birth weight of boys from OS pairs has generally been ascribed to a more successful *in utero* competition for nutrients of boys in the presence of a sister rather than a brother co-twin^17^. For girls, findings are less consistent. Girls from OS pairs were heavier at birth than those from SS pairs in Canadian twins^18^; similar but non-significant differences were observed in other populations^13,15,19^. Since androgens have shown to exert a positive effect on fetal growth^20^, a so-called twin testosterone transfer (TTT) hypothesis has been proposed^15^, by which females who develop with a male co-twin are potentially exposed to higher levels of prenatal testosterone (the most potent androgen) than females who develop with a female co-twin. This would then explain the greater birth weight in girls from OS than from SS pairs. However, other studies have reported roughly similar mean birth weight in girls from SS and OS pairs^11,12^ and, although non-significant, greater birth weight in SS twins^14^. Part of the discrepancy between findings might be explained by the different criteria used to select SS twins in the studies (all SS twins (monozygotic and dizygotic), SS dichorionic twins or only SS dizygotic twins).

According to studies in singletons showing that mean duration of gestation is shorter in boys than in girls^21,22^, there is evidence that boy-boy pairs have a shorter gestation than boy-girl pairs and girl-girl pairs; however, it is not clear whether gestational age differs between boy-girl pairs and girl-girl pairs^11,12,16^. Loos *et al*.^12^ observed that when controlling for the length of gestation, the birth weight differences between boys from SS and boys from OS pairs attenuated, suggesting that gestational age has an important role and that boys from OS pairs benefited from the slightly longer gestation. This study^12^ additionally showed that gestational age difference between boys and girls is smaller with greater birth weight, which confirmed the findings in singletons^22^.

Although birth weight is the most widely used measure of birth size in epidemiological studies^23^, alternative measures, such as birth length and ponderal index (PI), have also been of interest. PI is a measure of relative weight (assessed as birth weight per birth length cubed) that is more appropriate for newborns than body mass index. For example, short birth length has been found to be associated with adult all-cause mortality^24,25^ and greater PI with a higher risk of breast cancer mortality^26^. Birth length is also greater in boys than in girls^6^, but only one study compared SS and OS twins showing no differences^18^. Using only dizygotic (SS and OS) twins from 21 cohorts in 15 countries, we conducted an individual-based analysis of pooled twin cohorts (i) to analyze the association of co-twin’s sex with birth weight, length and PI, (ii) to ascertain whether gestational age differs between the three twin pair types (boy-boy, boy-girl and girl-girl) and (iii) to examine whether gestational age plays a role in the association between co-twin’s sex and the three indicators of birth size.

## Material and Methods

### Ethics

All participants were volunteers and they or their parents gave informed consent when participating in their original studies. No experimental data were asked and thus we did not ask ethical approval. Only a limited set of observational variables and anonymized data were delivered to the data management center at University of Helsinki. The pooled analysis was approved by the ethical committee of Department of Public Health, University of Helsinki, and the methods were carried out in accordance with the approved guidelines.

### Sample

This study is based on the data from the COllaborative project of Development of Anthropometrical measures in Twins (CODATwins), which was intended to pool data from all twin projects in the world having information on height and weight^27^. Information on birth weight of both SS and OS dizygotic twins was available for 21 twin cohorts from 15 countries (OS/SS ratio range = 0.3–2.2). Birth length and gestational age were available in 12 and 13 of these cohorts, respectively. The participating twin cohorts are identified in Table 1 (footnote) and were previously described in detail^27^.

**Table 1:** Descriptive statistics and regression coefficients for the difference in birth size of dizygotic twin boys and girls according to the sex of their co-twin (reference group same-sex dizygotic twins).

	SS			OS			B	p-value	CIs	
	n	Mean	SD	n	Mean	SD				
**Boys**										
Birth weight (kg)	17584	2.578	0.57	16417	2.608	0.56	0.031	<0.001	0.018	0.045
Birth length (cm)	8702	47.42	3.28	8170	47.60	3.30	0.160	0.006	0.045	0.274
PI at birth (kg/m^3^)	8702	24.41	3.12	8170	24.44	3.13	0.021	0.691	−0.083	0.125
**Girls**										
Birth weight (kg)	17432	2.495	0.54	16417	2.497	0.54	0.005	0.466	−0.008	0.018
Birth length (cm)	8128	46.94	3.14	8170	46.85	3.28	−0.089	0.125	−0.202	0.025
PI at birth (kg/m^3^)	8128	24.45	3.21	8170	24.46	3.18	0.005	0.933	−0.103	0.113

Participating twin cohorts in this study: Australian Twin Registry, Carolina African American Twin Study of Aging, Child and Adolescent Twin Study in Sweden, East Flanders Prospective Twin Survey, Finntwin12, Finntwin16, Gemini Study, Guinea-Bissau Twin Study, Hungarian Twin Registry, Italian Twin Registry, Japanese Twin Cohort, Longitudinal Israeli Study of Twins, Michigan Twins Study, Minnesota Twin Registry, Mongolian Twin Registry, Peri/Postnatal Epigenetic Twins Study, Qingdao Twin Registry of Children, Quebec Newborn Twin Study, Twins Early Developmental Study, West Japan Twins and Higher Order Multiple Births Registry and Young Netherlands Twin Registry.

**Figure 1:**
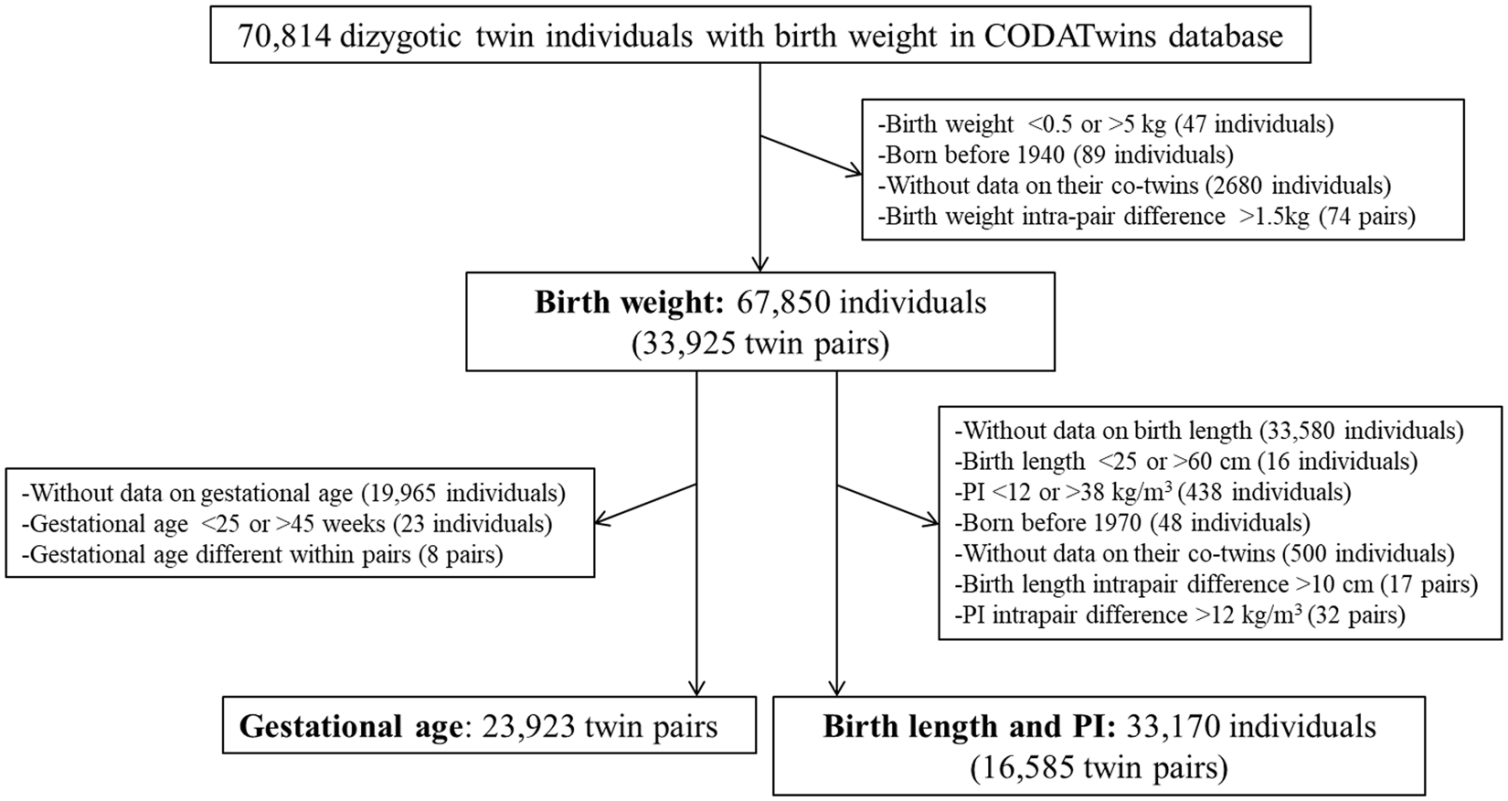
Flow chart of sample selection in the study. CODATwins: COllaborative project of Development of Anthropometrical measures in Twins; individuals: twin individuals; pairs: twin pairs.

After excluding monozygotic twins (n = 39,590), we had 70,814 dizygotic twin individuals with information on birth weight (Fig. 1). We removed individuals with birth weight <0.5 or >5 kg, or born before 1940 because there were too few of them for meaningful analyses. We next excluded individuals without data on their co-twins or with intra-pair difference in birth weight >1.5 kg leaving 67,850 individuals (33,925 complete twin pairs; 52% SS and 48% OS). For the analyses on birth length and PI [weight (kg)/height (m^3^)], we additionally excluded individuals without data on birth length, with birth length <25 or >60 cm, PI <12 or >38 kg/m^3^, intrapair difference in birth length >10 cm or PI >12 kg/m^3^, or born before 1970 leaving 33,170 individuals (16,585 complete twin pairs; 51% SS and 49% OS). These cut-off limits were defined based on the visual inspection of histograms for both birth size measures and intrapair differences in birth size.

For the analyses involving gestational age, from the 67,850 individuals with information on birth weight, we excluded twin individuals without data on gestational age and gestational age <25 or >45 weeks resulting in 23,923 twin pairs. Gestational age was defined as a categorical variable indicating completed weeks of gestation. We visually inspected histograms for each gestational week and removed unrealistic values for birth weight, length and PI for a given gestation (<0.2% for birth weight and <0.4% for birth length and PI). We further calculated birth weight, length and PI standardized by gestational age. These three measures of size at birth were expressed as SD scores of the respective means/weeks of gestation (z-scores; i.e., mean = 0 and SD = 1) to estimate their relative value for a given gestational age. Finally, birth weight standardized by gestational age was available for 23,870 twin pairs and birth length and PI standardized by gestational age for 14,890 twin pairs.

### Statistical analyses

Statistical analyses were conducted using the Stata statistical software package (version 12.0; StataCorp, College Station, Texas, USA). To compare the birth size (both unstandardized and standardized by gestational age) of individuals from OS pairs with that of individuals from SS pairs, we used linear regression models (twin type was used as the explanatory variable and birth size measures as the outcomes) adjusted for birth year and twin cohort separately by sex. The non-independence within twin pairs was taken into account by using the “cluster” option available in Stata. The association between twin pair type (boy-boy, boy-girl and girl-girl) and gestational age was analyzed using linear regression models (twin pair type was used as the explanatory variable and gestational age as the outcome variable) adjusted for birth year and twin cohort. To calculate the odds ratios (OR) for the risk of delivery at less than 37, 34 and 31 weeks (“preterm delivery” will be used as general term) between the three twin pair types, we used logistic regression models adjusted for birth year and twin cohort.

Finally, we compared gestational age between boys and girls separately by 7 birth weight classes of 0.5 kg (0.75–1.25, …, 3.75–4.25 kg) and 5 birth length classes of 5 cm (32.5–37.5, …, 52.5–57.5 cm). For these analyses, in addition to the exclusion of unrealistic birth size values for a given gestational age, we removed birth weight values <0.75 and ≥4.25 (n = 54) and birth length values <32.5 and ≥57.5 (n = 39) because the sample size was too small to create more categories. Linear regression models (sex was used as the explanatory variable and gestational age as the outcome variable) adjusted for birth year, twin cohort and non-independence within twin pairs were used.

## Results

Descriptive statistics for the three birth size measures of twin individuals in relation to the sex of their co-twin are presented in Table 1. The four twin type and sex groups had similar sample sizes ranging from 16,417 to 17,584 for birth weight and from 8,128 to 8,702 for birth length and PI. As expected, mean birth weight and length were greater in boys than in girls, with a difference of 83 g and 0.48 cm in SS twins and 111 g and 0.75 cm in OS twins, whereas PI was very similar in both sexes. Boys having a co-twin sister were, on average, 31 g (95% CI 18 to 45, p < 0.001) heavier and 0.16 cm (95% CI 0.045 to 0.274, p = 0.006) longer than those with a co-twin brother; however, PI at birth was similar in SS and OS twins. In girls, the sex of the co-twin was not associated with birth size.

We then compared mean gestational age and the risk of preterm delivery between twin pair types (boy-boy, boy-girl and girl-girl) (Table 2). Compared to boy-boy pairs, mean gestational age was greater in both boy-girl [0.13 weeks; 95% CI 0.06 to 0.21 (p = 0.001)] and girl-girl [(0.20 weeks; 95% CI 0.11 to 0.28 (p < 0.001)] pairs. In additional analyses using girl-girl pairs as the reference group (results not shown), gestational age was not significantly different between girl-girl and boy-girl pairs [−0.06 weeks; 95% CI −0.14 to 0.02 (p = 0.121)]. Further, both boy-girl and girl-girl pairs showed a lower risk (OR) of delivery before 37 weeks [0.92; 95% CI 0.86 to 0.98 (p = 0.007) and 0.88; 95% CI 0.82 to 0.95 (p = 0.001), respectively] and 34 weeks [0.89; 95% CI 0.81 to 0.99 (p = 0.024) and 0.80; 95% CI 0.71 to 0.90 (p = <0.001), respectively] than boy-boy pairs; a similar trend was observed for the risk before 31 weeks but did not reach statistical significance [0.88; 95% CI 0.73 to 1.06 (p = 0.165) and 0.82; 95% CI 0.66 to 1.02 (p = 0.078), respectively)]. Using girl-girl pairs as the reference group, boy-girl pairs showed slightly higher risk of delivery before 34 weeks [1.12; 95% CI 1.01 to 1.24 (p = 0.036)] than girl-girl pairs, but not before 37 weeks [1.04; 95% CI 0.97 to 1.11 (p = 0.291)] or 31 weeks [1.07; 95% CI 0.88 to 1.31 (p = 0.510)].

**Table 2:** Gestational age and risk of preterm delivery by dizygotic twin pair type (reference group boy-boy pairs).

	Boy-boy pairs	Boy-girl pairs	RC^a^/OR^b^ (95% CIs)	p-value	Girl-girl pairs	RC^a^/OR^b^ (95% CIs)	p-value
	n = 6204	n = 11,660			n = 6059		
Gestational age (SD) weeks	36.63 ± 2.57	36.74 ± 2.51	0.13(0.06, 0.21)^a^	0.001	36.87 ± 2.48	0.20(0.11, 0.28)^a^	<0.001
Delivery before 37 weeks	2383 (38.4)	4274(36.7)	0.92(0.86, 0.98)^b^	0.007	2134(35.2)	0.88(0.82, 0.95)^b^	0.001
Delivery before 34 weeks	712(11.5)	1223(10.5)	0.89(0.81, 0.99)^b^	0.024	564(9.3)	0.80(0.71, 0.90)^b^	<0.001
Delivery before 31 weeks	182(3.0)	305(2.7)	0.88(0.73, 1.06)^b^	0.165	145(2.5)	0.82(0.66, 1.02)^b^	0.078

Data are mean ± standard deviation or number of pairs (%).

^a^Regression coefficient (RC) for the difference in gestational age.

^b^Odds ratios (OR) for the risk of preterm delivery.

In Table 3, we present the descriptive statistics and regression coefficients for the difference in gestational age standardized birth size measures of twins in relation to the sex of their co-twin. Birth weight was 0.028 z-score greater in OS than in SS twins of both sexes, that is, standardization by gestational age attenuated the magnitude of the association to half of the unstandardized value in boys and created a positive difference in girls. When regression coefficients were back-transformed to original values, maximum differences of 15 g in boys and 12 g in girls were observed. For birth length, the association was significant only in boys and the attenuation after the standardization by gestational age was modest; boys from OS pairs had 0.052 z-score (0.11–0.16 cm depending on gestational age) greater length than boys from SS pairs. In agreement with the unstandardized results, PI was not associated with the sex of the co-twin.

**Table 3:** Descriptive statistics and regression coefficients for the difference in gestational age standardized birth size of dizygotic twin boys and girls according to the sex of their co-twin (reference group same-sex dizygotic twins).

	SS			OS			B	p-value	CIs	
	n	Mean	SD	n	Mean	SD				
**Boys**										
Birth weight (z-score)	12384	−0.027	1.00	11628	0.029	0.99	0.060	<0.001	0.031	0.089
Birth weight for gestational age (z-score)		−0.015	1.00		0.016	1.00	0.028	0.044	0.001	0.055
Birth length (z-score)	7776	−0.030	0.99	7379	0.032	1.00	0.062	0.001	0.025	0.098
Birth length for gestational age (z-score)		−0.025	0.98		0.027	1.01	0.052	0.004	0.017	0.088
PI at birth (z-score)	7776	−0.001	0.99	7379	0.001	1.01	0.001	0.955	−0.034	0.036
PI at birth for gestational age (z-score)		0.003	0.99		−0.004	1.01	−0.007	0.679	−0.042	0.027
**Girls**										
Birth weight (z-score)	12100	0.004	0.99	11628	−0.004	1.01	0.003	0.818	−0.026	0.033
Birth weight for gestational age (z-score)		−0.016	1.00		0.017	1.00	0.028	0.050	0.000	0.055
Birth length (z-score)	7246	0.015	0.98	7379	−0.015	1.02	−0.026	0.174	−0.063	0.011
Birth length for gestational age (z-score)		−0.004	1.00		0.004	1.00	0.012	0.504	−0.024	0.049
PI at birth (z-score)	7246	0.001	1.00	7379	−0.001	1.00	−0.002	0.931	−0.037	0.034
PI at birth for gestational age (z-score)		−0.002	1.00		0.003	1.00	0.005	0.782	−0.030	0.040

**Figure 2:**
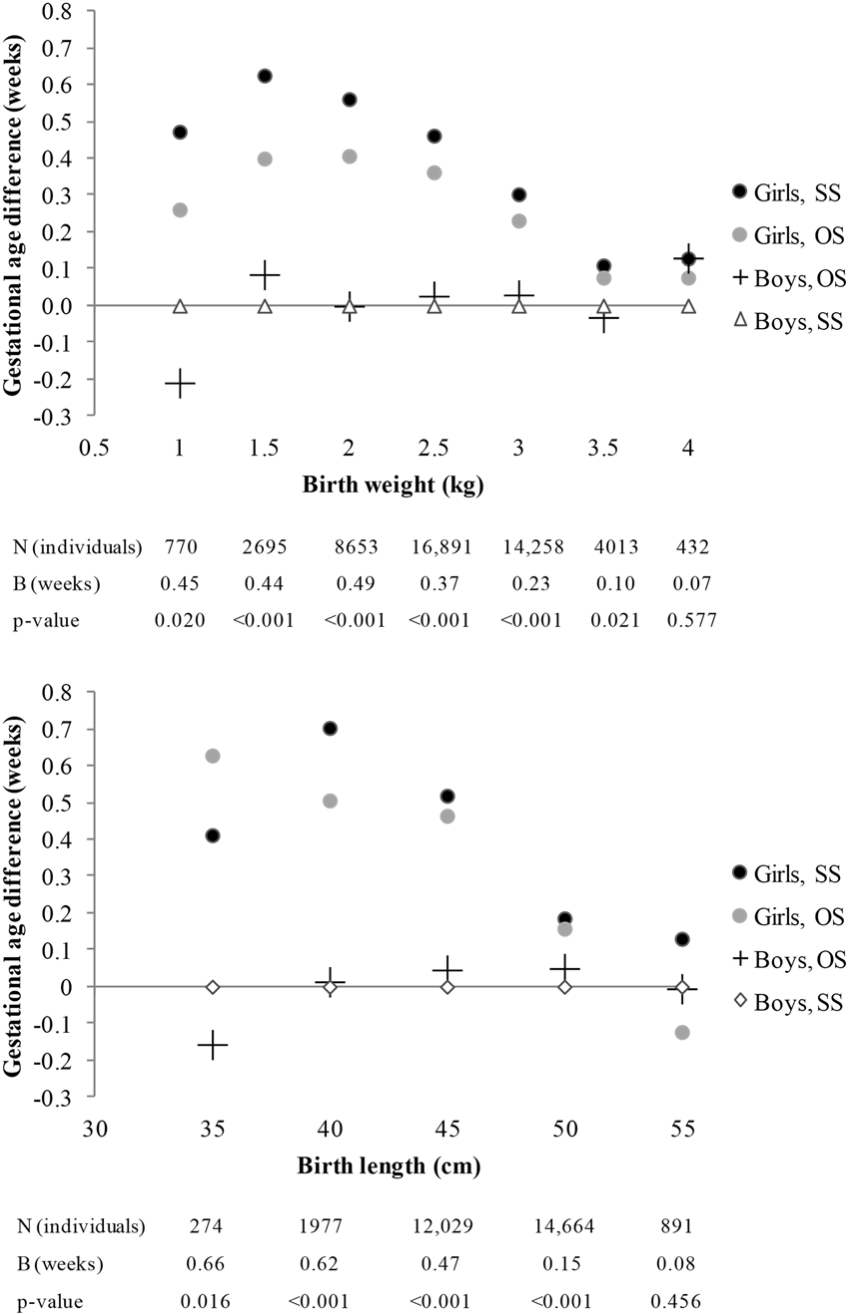
Difference in gestational age (weeks) between individuals of the four twin type groups. Indicated weights and lengths are midpoint values of birth weight classes of 0.5 kg and 5 cm, respectively. B: regression coefficient for the difference in gestational age between sexes.

Figure 2 shows, per birth weight and length class, the difference of gestational age between boys from SS pairs (reference group) and boys from OS pairs, girls from OS pairs, and girls from SS pairs. For any given birth weight and length class, the gestational age of boys from SS pairs and boys from OS pairs was very similar. Gestation of girls was longer than that of boys for birth weights ranging between 0.75 kg and 3.75 kg and birth lengths ranging between 32.5 and 52.5 cm. The difference in gestational age between boys and girls was greatest for the smaller birth size classes and decreased with increasing birth weight and length from 2.5 kg and 45 cm classes.

## Discussion

The present study, based on a multinational database of 21 twin cohorts from 15 countries, showed that birth weight and length are associated with the sex of the co-twin in boys. Differences in birth size between twins from SS and OS dizygotic twin pairs were of small magnitude and partly explained by differences in gestational age between boys and girls. Our results thus support the role of gestational age in the associations between co-twin’s sex and birth size and refine previous findings by considering, in addition to birth weight, also birth length and PI.

Boys with a co-twin sister were, on average, 31 g heavier at birth than those with a co-twin brother. Our findings are in line with previous studies showing a difference of 57 g^10^, 64 g^11^ and 78 g^12^, even when different inclusion criteria for SS twins were used. For girls, birth weight was very similar in SS and OS twins, which is also in agreement with these studies^10–12^. Boys with a co-twin sister were 0.16 cm longer than those with a co-twin brother, but differences were not significant in girls. Similar birth length in girls from SS and OS pairs was also observed in Canadian twins^18^. The lack of association of PI at birth with the sex of the co-twin may be because PI is a mathematical ratio of weight-to-length and proportional reduction in both birth weight and birth length will keep the ratio fairly unaffected. The attenuation of the associations between birth weight and co-twin’s sex after controlling for gestational age in boys was also observed in the study of Belgian twins^12^ (a part of this sample is included in the CODATwins project as the East Flanders Prospective Twin Survey), and has been attributed to a longer gestation in the presence of a co-twin sister.

Although Luke *et al*.^16^ found longer mean gestational age in OS pairs than in SS pairs in both sexes, our findings of shortest gestations in boy-boy pairs, intermediate in boy-girl pairs and longest in girl-girl pairs (36.6, 36.7 and 36.9 weeks, respectively) are in line with those observed in Belgian^12^ (36.4, 36.8 and 36.9 weeks, respectively) and Israeli^11^ (34.8, 35.1 and 35.2 weeks, respectively) twins. In the present study, the mean gestational ages between boy-girl and girl-girl pairs were not significantly different. Loos *et al*.^12^ reported a similar pattern for gestational age and concluded that it is the girl who governs the length of gestation, in such a way that there is a female-protective factor that prolongs gestation. However, Melamed *et al*.^11^ observed that mean gestational age and the risk of delivery before 31 and 28 weeks were significantly different between boy-girl and girl-girl pairs and they suggested that a potential “dose-dependent male-offending factor” might increase the risk of prematurity. Since in this multinational study the risk of delivery before 34 weeks was lower in girl-girl than in boy-girl pairs, this interpretation concerning a “dose-dependent male-offending factor” could also be applied to our results.

Although elucidating the mechanisms behind the association between the sex of the co-twin and birth size was not an objective of the present study, our results are opposite in direction to that predicted by the TTT hypothesis, at least in boys. We showed that gestational age has a role in the association between the co-twin’s sex and birth size. Melamed *et al*.^11^ speculated that the shortest gestational age in boy-boy pairs might be explained by the higher level of androgens in pregnancies with boys, which has been implicated in the onset of preterm labor^28^. Moreover, we do not know by which mechanisms, even after adjustment for gestational age, boys from OS pairs are significantly heavier and longer than boys from SS pairs. It has been suggested that, if the amount of nutrient available in boy-boy and boy-girl pregnancies are equal, boys in boy-girl pregnancies compared with those in boy-boy pregnancies will be more successful in the competition for nutrient because girls are programmed to grow slower^29^. However, girls from OS pairs were slightly heavier than those from SS pairs (after adjustment for gestational age), which is in opposite direction to the hypothesis of such maternal constraint. Moreover, and in agreement with previous studies in singletons^22^ and twins^12^, we showed that, to attain a given birth weight and length, the gestation of boys is shorter than that of girls, and the gestational age differences between boys and girls are smallest for the heaviest and longest children, which is not in accordance with this hypothesis either. This suggests that boys grow at a faster pace than girls, but that girls catch-up late in gestation. Pergament *et al*.^30^ found that female embryos, compared to male embryos, are already delayed in early embryonic development. It has been speculated that some critical time windows of development may be slightly different for boys and girls, and that this phenomenon may be one of the reasons for sex differences in sensitivity to fetal programming. In order to shed new light into the mechanisms behind the association between birth size and the co-twin’s sex, an interesting starting point for future studies would be to collect measures of fetal growth by ultrasound imaging at different stages of pregnancy to elucidate when these differences emerge.

The main strength of the present study is the large sample size of our multinational database of twin cohorts with information on birth weight, length and gestational age. We performed an individual-based pooled analysis to provide results for this sample including the large majority of existing twin cohorts having birth related measures. Generalization for the global population is, however, not possible because countries or regions are not equally represented and the database is heavily weighted towards European ancestry populations following westernized lifestyle. Another limitation of the data is that most of the measures were parentally reported^27^. However, the accuracy between reported birth weights and medical records of birth weights (in singletons) reached high kappa values (~0.90)^31,32^. Zygosity was also self-reported and not verified by DNA testing in the majority of studies. It is thus possible that misclassification of a small fraction of MZ twins as SS dizygotic twins could have contributed to part of the observed small differences in birth size between twin type groups. Finally, distinction between spontaneous and iatrogenic preterm birth (e.g. by caesarean section) is lacking.

We conclude from this multinational study that boys with a co-twin sister are heavier and longer at birth than those with a co-twin brother, but birth size in girls is not associated with the co-twin’s sex. The differences in boys are modest and partly explained by a longer gestation in the presence of a co-twin sister. Boy-boy pairs have a shorter length of gestation and a higher risk of preterm delivery than boy-girl and girl-girl pairs, and the difference in gestational age between boys and girls was generally smaller with greater birth size. Although the effects are too small to be of clinical significance, these findings have theoretical significance and might help to shed light on the underlying mechanisms linking birth size and the sex of the co-twin in future research.

## References

[1] Wilcox AJ (2001). On the importance–and the unimportance–of birthweight. Int. J. Epidemiol..

[2] Risnes KR (2011). Birthweight and mortality in adulthood: a systematic review and meta-analysis. Int. J. Epidemiol..

[3] Williams RL (1982). Fetal growth and perinatal viability in California. Obstet. Gynecol..

[4] Cogswell ME, Yip R (1995). The influence of fetal and maternal factors on the distribution of birthweight. Semin. Perinatol..

[5] Glinianaia SV, Skjaerven R, Magnus P (2000). Birthweight percentiles by gestational age in multiple births. A population-based study of Norwegian twins and triplets. Acta Obstet. Gynecol. Scand..

[6] Sankilampi U, Hannila ML, Saari A, Gissler M, Dunkel L (2013). New population-based references for birth weight, length, and head circumference in singletons and twins from 23 to 43 gestation weeks. Ann. Med..

[7] Li Z, Umstad MP, Hilder L, Xu F, Sullivan EA (2015). Australian national birthweight percentiles by sex and gestational age for twins, 2001-2010. BMC Pediatr..

[8] Imaizumi Y (2003). A comparative study of zygotic twinning and triplet rates in eight countries, 1972-1999. J. Biosoc. Sci..

[9] Blickstein, I., Keith, L.G., Keith, D.M (eds). *Multiple Pregnancy*, 2nd ed. Taylor and Francis Group (2005).

[10] Goldman RD, Blumrozen E, Blickstein I (2003). The influence of a male twin on birthweight of its female co-twin - a population-based study. Twin Res..

[11] Melamed N, Yogev Y, Glezerman M (2009). Effect of fetal sex on pregnancy outcome in twin pregnancies. Obstet. Gynecol..

[12] Loos RJ, Derom C, Eeckels R, Derom R, Vlietinck R (2001). Length of gestation and birthweight in dizygotic twins. Lancet.

[13] Treloar, S. A. & Whitfiled, J. B. Birthweights in same-sex and opposite-sex twin pregnancies (Letter to editor). *Twin Res.* **5**, 310 (2002).

[14] Orlebeke JF, van Baal GC, Boomsma DI, Neeleman D (1993). Birth weight in opposite sex twins as compared to same sex dizygotic twins. Eur. J. Obstet. Gynecol. Reprod. Biol..

[15] Glinianaia SV, Magnus P, Harris JR, Tambs K (1998). Is there a consequence for fetal growth of having an unlike-sexed cohabitant in utero?. Int. J. Epidemiol..

[16] Luke B (2005). Gender mix in twins and fetal growth, length of gestation and adult cancer risk. Paediatr. Perinat. Epidemiol..

[17] James WH (2002). Birthweight in dizygotic twins. Twin Res..

[18] Jahanfar S, Lim K (2016). The Impact of Gender on Anthropometric Measures of Twins. Twin Res. Hum. Genet..

[19] Alexanderson C, Henningsson S, Lichtenstein P, Holmang A, Eriksson E (2011). Influence of having a male twin on body mass index and risk for dyslipidemia in middle-aged and old women. Int. J. Obes. (Lond).

[20] de Zegher F (1998). Androgens and fetal growth. Horm. Res..

[21] Melamed N, Yogev Y, Glezerman M (2010). Fetal gender and pregnancy outcome. J. Matern. Fetal. Neonatal Med..

[22] de Zegher F, Devlieger H, Eeckels R (1999). Fetal growth: boys before girls. Horm. Res..

[23] Silventoinen, K. Children’s anthropometrics and later disease incidence in *The Oxford Handbook of Economics and Human Biology* (eds Komlos, J. & Kelly, I. R.). Oxford University Press, pp 604–620 (2015).

[24] Andersen AM, Osler M (2004). Birth dimensions, parental mortality, and mortality in early adult age: a cohort study of Danish men born in 1953. Int. J. Epidemiol..

[25] Kajantie E (2005). Size at birth as a predictor of mortality in adulthood: a follow-up of 350 000 person-years. Int. J. Epidemiol..

[26] Sovio U, Jones R, Dos Santos Silva I, Koupil I (2013). Birth size and survival in breast cancer patients from the Uppsala Birth Cohort Study. Cancer Causes Control.

[27] Silventoinen K (2015). The CODATwins Project: The Cohort Description of Collaborative Project of Development of Anthropometrical Measures in Twins to Study Macro-Environmental Variation in Genetic and Environmental Effects on Anthropometric Traits. Twin Res. Hum. Genet..

[28] Makieva S, Saunders PT, Norman JE (2014). Androgens in pregnancy: roles in parturition. Hum. Reprod. Update.

[29] James WH (2002). Gestation and birthweight in dizygotic twins. Lancet.

[30] Pergament E, Fiddler M, Cho N, Johnson D, Holmgren WJ (1994). Sexual differentiation and preimplantation cell growth. Hum. Reprod..

[31] McCormick MC, Brooks-Gunn J (1999). Concurrent child health status and maternal recall of events in infancy. Pediatrics.

[32] Jensen CB, Gamborg M, Heitmann B, Sorensen TI, Baker JL (2015). Comparison of birth weight between school health records and medical birth records in Denmark: determinants of discrepancies. BMJ Open.

